# Fibroblast derived C3 promotes the progression of experimental periodontitis through macrophage M1 polarization and osteoclast differentiation

**DOI:** 10.1038/s41368-025-00361-z

**Published:** 2025-04-17

**Authors:** Feilong Ren, Shize Zheng, Huanyu Luo, Xiaoyi Yu, Xianjing Li, Shaoyi Song, Wenhuan Bu, Hongchen Sun

**Affiliations:** 1 Hospital of Stomatology, Jilin University, Changchun, China; 2 Jilin Provincial Key Laboratory of Tooth Development and Bone Remodeling, School and Hospital of Stomatology, Jilin University, Changchun, China; 3 Jilin Provincial Key Laboratory Oral Biomedical Engineering, Jilin University, Changchun, China

**Keywords:** Mechanisms of disease, Proteolysis

## Abstract

Complement C3 plays a critical role in periodontitis. However, its source, role and underlying mechanisms remain unclear. In our study, by analyzing single-cell sequencing data from mouse model of periodontitis, we identified that C3 is primarily derived from periodontal fibroblasts. Subsequently, we demonstrated that C3a has a detrimental effect in ligature-induced periodontitis. *C3ar*^*−/−*^ mice exhibited significantly less destruction of periodontal support tissues compared to wild-type mice, characterized by mild gingival tissue damage and reduced alveolar bone loss. This reduction was associated with decreased production of pro-inflammatory mediators and reduced osteoclast infiltration in the periodontal tissues. Mechanistic studies suggested that C3a could promote macrophage polarization and osteoclast differentiation. Finally, by analyzing single-cell sequencing data from the periodontal tissues of patients with periodontitis, we found that the results observed in mice were consistent with human data. Therefore, our findings clearly demonstrate the destructive role of fibroblast-derived C3 in ligature-induced periodontitis, driven by macrophage M1 polarization and osteoclast differentiation. These data strongly support the feasibility of C3a-targeted interventions for the treatment of human periodontitis.

## Introduction

Periodontitis is a chronic, progressive and destructive disease of the periodontal supporting tissues (gingiva, periodontal ligament, alveolar bone and cementum), initiated primarily by dental plaque.^1^ Epidemiological studies indicate that periodontitis is one of the most prevalent chronic inflammatory diseases worldwide, affecting nearly all age groups, with approximately 700 million people suffering from various degrees of periodontitis globally.^2,3^ Periodontitis leads to inflammation of the periodontal soft tissues, loss of periodontal attachment and alveolar bone resorption, which can result in tooth mobility, displacement and even loss.^4^ Furthermore, periodontitis is closely associated with the development of other diseases, such as cardiovascular diseases, respiratory diseases, Alzheimer’s disease, adverse pregnancy outcomes, rheumatoid arthritis, kidney diseases and diabetes.^5–11^ Therefore, periodontitis is a critical disease affecting both oral and systemic health, imposing a substantial economic burden on both the nation and individuals. The treatment of periodontitis is a complex and multi-layered process aimed at eliminating periodontal plaque microorganisms as much as possible, reducing periodontal tissue inflammation and achieving periodontal tissue repair and regeneration.^12^ Although with ongoing advancements in clinical practice and basic research, therapies transit from traditional treatment methods including pharmacotherapy and basic therapy to a series of emerging treatment methods, such as periodontal surgery, guided tissue regeneration, root planing, enamel matrix derivative compounds, and stem cell tissue engineering techniques.^13,14^ They can only improve the degree of periodontal inflammation and reduce the difficulty of alveolar bone repair to a certain extent but still fail to achieve satisfactory therapeutic outcomes. Therefore, exploring new therapeutic strategies or targets for periodontitis is urgently needed.

Studies have shown that the primary cause of periodontal tissue destruction is not the direct action of periodontal pathogens, but rather the excessive immune response of the host in defense against the invasion of these pathogens.^15^ Therefore, exploring in depth the immunopathological mechanisms of periodontitis may offer novel therapeutic targets for its treatment. As a crucial component of the immune system, the complement system plays a significant role in the development and progression of periodontitis.^16^ It is a key component of innate immunity, activated rapidly in response to infection or tissue stress. Complement activation generates a set of effector molecules with diverse biological functions. The small fragments (C3a, C5a) mediate inflammation by interacting with their specific receptors (C3aR, C5aR); the large fragment C3b mediates opsonization through ligation of C3b receptors (e.g., CR3) and the terminal product C5b-9 mediates direct killing of pathogens. Both C3a and C5a are described as important inflammatory mediators.^17^ Many studies have shown that the C3a primarily exerts pro-inflammatory effects. For example, research has demonstrated that inflammatory tissue priming relies on the intracellular activation of complement C3 and C3a receptors in synovial fibroblasts.^18^ Additionally, in a mouse model of unilateral ureteral obstruction, complement C3 exacerbates renal interstitial fibrosis by facilitating the M1 macrophage phenotype.^19^ However, increasing evidence suggests that the C3a exhibits anti-inflammatory properties. The C3a have been shown to exert anti-inflammatory role in several disease models, such as endotoxin shock and systemic lupus erythematosus.^20^ These findings imply that the role of C3a in periodontitis is ambiguous. Most importantly, the origin and the mechanisms by which C3a contributes to the pathogenesis of periodontitis remain unclear. Therefore, elucidating the source, role and mechanism of the C3a in the development of periodontitis is of critical importance.

Except for the complement system, macrophages are essential parts of the innate immune system, which identify, phagocytose, remove bacteria and foreign bodies.^21^ Functionally, macrophages are divided into typical (M1), nontypical (M2) and intermediate (M0) types in local tissue microenvironments. As the first line of defense against periodontal pathogens, macrophages can kill pathogenic bacteria through their powerful bactericidal phagocytosis. However, their excessive activation into M1 leads to the destruction of periodontal tissues and aggravates the process of periodontitis.^22^ Indeed, many M1 macrophage derived inflammatory factors such as interleukin-1 (IL-1) and tumor necrosis factor-*α* (TNF-*α*) were detected in bone destruction area of periodontitis.^23^ It is also shown that the amounts of M1 macrophages are significantly increased compared with M2 macrophages in bone resorption areas of mice with periodontitis, which indicates that there is an imbalance between M1 and M2 macrophages in periodontitis, leading to the dominant position of M1 macrophages and subsequently alveolar bone resorption.^24^ Therefore, it is of great significance for chronic inflammation elimination by reversing macrophage polarization and increasing M2 macrophages. Researchers have explored the treatment of periodontitis by promoting M2 polarization of macrophages through the inhibition of the Akt2/JNK/c-Jun and Akt2/miR-155-5p/DET1/c-Jun signaling pathways.^25^ Additionally, some studies have investigated increasing the number of M2 macrophages by delivering microparticles carrying CCL2 as a therapeutic strategy for periodontitis.^26^ Given that macrophages highly express the C3aR, it is reasonably hypothesized that the C3a promotes periodontal tissue inflammation in periodontitis mice by mediating the polarization of macrophages.

The primary pathological feature of periodontitis is the destruction of alveolar bone, which is primarily mediated by osteoclasts.^27^ Osteoclasts are giant, multinucleated, non-proliferative bone-resorbing poly karyons formed by the differentiation and fusion of hematopoietic precursors from the monocytic and macrophagic lineage.^28^ The induction of osteoclast lineage is driven by two essential cytokines: macrophage colony-stimulating factor (M-CSF) and receptor activator of nuclear factor-kappa B ligand (RANKL).^29^ These and other pro-osteoclastogenic factors are locally produced by osteoblasts and other cells in the bone microenvironment. Given that myeloid cells are the precursors of osteoclasts and are the primary cell type expressing C3aR, it is hypothesized that the C3a promotes alveolar bone resorption in periodontitis mice by facilitate osteoclast differentiation.

In this study, we established a periodontitis model using transgenic mice with a deletion of the C3a receptor, C3aR. Micro-computed tomography (Micro-CT) was employed to assess alveolar bone resorption in the mice, while hematoxylin and eosin (HE) and Masson staining were used to observe destruction of the periodontal tissues. Immunohistochemical staining was performed to detect the expression of inflammatory cytokines in the periodontal tissues. Single-cell sequencing data of periodontal tissues revealed that fibroblasts are the primary source of C3, whereas myeloid cells predominantly express C3aR. Subsequently, through immunofluorescence staining, flow cytometry, and tartrate-resistant acid phosphatase (TRAP) staining, we demonstrated that fibroblasts derived C3 promotes the progression of periodontitis by enhancing M1 macrophage polarization and osteoclast differentiation. Finally, single-cell sequencing data from human periodontal tissues confirmed that these findings are also applicable to humans. Our research elucidates the role and mechanism of the C3a in chronic periodontitis, providing a theoretical basis and potential therapeutic targets for the prevention and treatment of periodontitis.

## Results

### The primary source of complement C3 during periodontitis in mice is periodontal fibroblasts

**Fig. 1:**
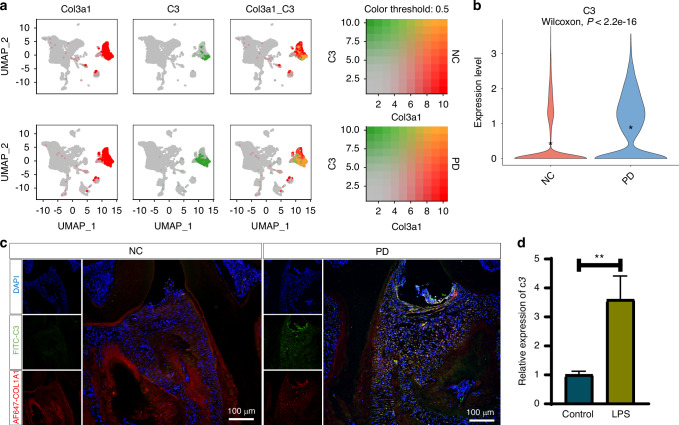
Periodontal fibroblasts are the main source of complement C3 in mice. **a** Analysis results of the co-expression of fibroblast marker *Col3a1* and complement *C3*. **b** Violin plot of the C3 gene between the NC and PD groups in mice. **c** Immunofluorescence co-staining results of fibroblast marker COL1A1 and complement C3 in healthy mouse periodontal tissue and periodontal tissue from mice with periodontitis. Scale bar 100um. **d** PCR results of *C3* gene expression in mouse gingival fibroblasts with or without LPS stimulation. Scale bar, 100 μm. Statistic are shown in mean ± SD (**d**) accessed by the unpaired *t* test. **P < 0.01

The central component of the complement system, C3, plays a pivotal role in the initial and progression of periodontitis. However, the specific cellular sources of complement C3 within periodontal tissues remain unclear. To address these questions unbiasedly, we downloaded single-cell transcriptomic sequencing data of periodontal tissues from normal and periodontitis mice from the GEO database. After quality control and dimensionality reduction, the entire cell population was initially classified into 8 different cell clusters based on classical cell type markers (Supplementary Fig. S1d), including epithelial cells, transit-amplifying cells (TAC), fibroblasts, endothelial cells, B/plasma cells, macrophages, pericytes and glia cells (Supplementary Fig. S1a and b). Subsequently, we compared the cluster proportional changes between the two groups. The results revealed that, compared to healthy mice, the proportions of endothelial cells, plasma cells, macrophages and fibroblasts significantly increased in the periodontal tissues of periodontitis mice, while epithelial cells and TAC significantly decreased. Pericytes and glia cells showed no significant changes, and there were no noticeable differences in cell subtypes between the two groups (Supplementary Fig. S1c). Finally, we examined the expression levels of C3 across the entire cell population. The findings indicated that fibroblasts are the primary cell type expressing C3 (Fig. 1a). In the periodontal microenvironment of periodontitis mice, the expression levels of C3 in fibroblasts were significantly elevated compared to healthy mice (Fig. 1b). Simultaneously, we measured the protein levels of complement C3a in the serum of healthy and periodontitis mice. Our results showed a significant increase in C3a levels in the serum of periodontitis mice compared to healthy mice (Supplementary Fig. S2a). At the same time, we validated in vivo, using immunofluorescence co-staining, that the fibroblast-derived C3 levels were higher in the periodontitis group compared to the normal group (Fig. 1c). Additionally, we verified in vitro through RT-qPCR that LPS stimulation increased the C3 gene expression in mouse gingival fibroblasts (Fig. 1d). Above results indicating that C3 is closely related to the development and progression of periodontitis.

### C3aR deficiency alleviated alveolar bone loss in mice with ligature-induced periodontitis

**Fig. 2:**
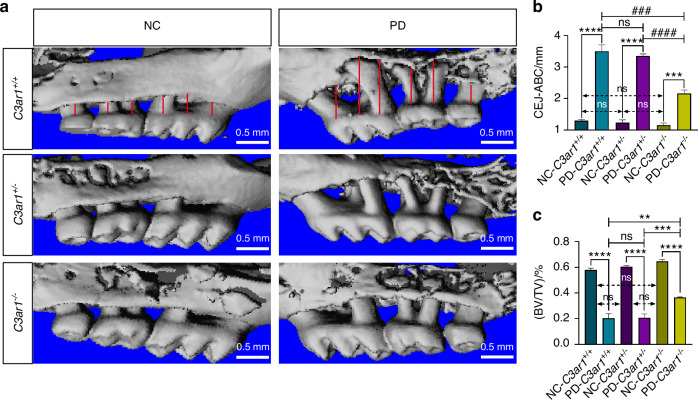
C3a leads to alveolar bone loss in periodontitis mice. **a** Reconstructed 3D micro-computed tomography images of upper molars. **b** Combined distance of the six indicated sites from the cementum-enamel junction (CEJ) to the alveolar bone crest (ABC) at the buccal surfaces. **c** Alveolar bone volume to tissue volume (BV/TV) between the first and second upper molars (%). PD periodontitis, NC normal control. Scale bar, 0.5 mm. Statistics are shown in mean ± SD (**b**, **c**) accessed by the unpaired *t* test. **P < 0.01; ***P < 0.001; ****P < 0.000 1; ^###^*P* < 0.001; ^####^*P* < 0.000 1; ns, nonsignificant, respectively

Complement C3 is hydrolyzed into C3a and C3b by the action of C3 convertase. C3b forms C5 convertase, contributing to the complement cascade, while C3a is released and plays a role in various disease processes. The role of complement C3a in periodontitis remains controversial. To address this issue, we established *C3ar1* knockout mice. After breeding and rearing, we obtained three genotypes of transgenic mice: *C3ar1*^*+/+*^, *C3ar1*^*+/−*^ and *C3ar1*^*−/−*^ (Supplementary Fig S3a). These mice were subsequently used to induce periodontitis models. After 8 days of ligature placement, micro-CT was employed to assess periodontal bone resorption. Quantitative measurements of the distance from the alveolar crest to the cementoenamel junction and bone volume fraction, along with three-dimensional reconstructions, revealed significant bone loss on ligatured sides compared to non-ligated sides across all genotypes, confirming successful establishment of the periodontitis model. Non-ligated sides showed no significant differences in alveolar bone levels among the three genotypes, indicating that *C3ar1* gene deletion does not affect healthy alveolar bone levels. Compared to *C3ar1*^*+/+*^ and *C3ar1*^*+/−*^ mice, *C3ar1*^*−/−*^ mice showed significantly less ligature-induced bone loss, suggesting that C3aR receptor deletion mitigates alveolar bone loss in periodontitis mice. Importantly, partial deletion of the *C3ar1* gene does not affect C3a receptor expression or the extent of alveolar bone loss, supported by observations of no significant difference in ligature-induced bone resorption between *C3ar1*^*+/+*^ and *C3ar1*^*+/−*^ mice (Fig. 2a–c).

### C3aR deficiency alleviated inflammatory cells infiltration and collagen fibers degradation in mice with ligature-induced periodontitis

**Fig. 3:**
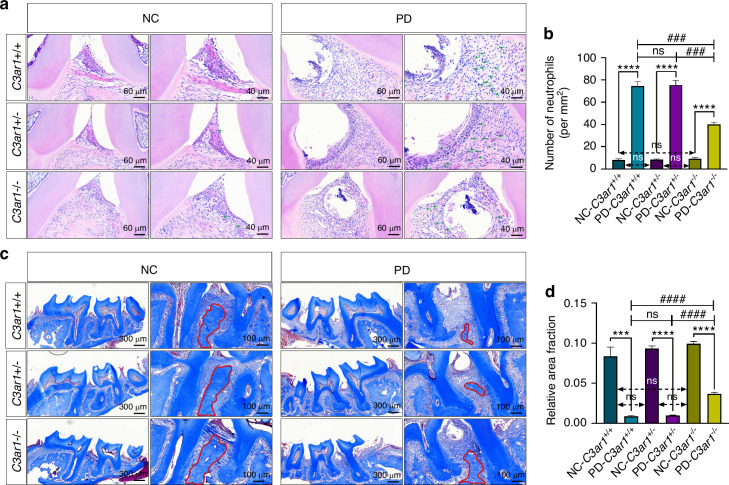
C3a induces neutrophil infiltration and collagen degradation in the periodontal tissues of periodontitis mice. **a** Hematoxylin-eosin staining of periodontal tissue between the first and second upper molars (green arrow indicates neutrophils). **b** Comparison of neutrophil numbers. **c** Masson’s trichrome staining of periodontal tissue sections between the first and second upper molars. **d** Comparison of the area fraction of collagen (indicated by the red curve). Scale bar, (HE) 60 μm and 40 μm, (Masson) 300 μm and 100 μm. Statistics are shown in mean ± SD (**b**, **d**) accessed by the unpaired *t* test. ***P < 0.001; ****P < 0.000 1; ^###^*P* < 0.001; ^####^*P* < 0.000 1; ns, nonsignificant, respectively

The severity of periodontitis manifests not only in alveolar bone resorption but also in epithelial disruption, subepithelial fibrous tissue inflammation characterized by neutrophil infiltration, and collagen fiber degradation.^30^ Therefore, we evaluated epithelial integrity and neutrophil infiltration between the first and second molars of periodontitis mice using HE staining. Our results indicate that, compared to the non-ligated side, all three genotypes of ligated mice exhibited loss of normal gingival papilla morphology, varying degrees of epithelial integrity disruption and increased neutrophil infiltration in the subepithelial connective tissue. Non-ligated sides showed no significant differences in gingival papilla morphology, epithelial integrity or neutrophil infiltration levels among the three genotypes. Compared to *C3ar1*^*+/+*^ and *C3ar1*^*+/−*^ mice, *C3ar1−/−* mice retained gingival papilla morphology, relative epithelial integrity and significantly reduced neutrophil infiltration in the ligated side, with no significant differences between *C3ar1*^*+/+*^ and *C3ar1*^*+/−*^ mice, indicating that C3aR deletion alleviates periodontal soft tissue destruction and inflammatory cell infiltration (Green arrow) in periodontitis mice (Fig. 3a, b). Subsequently, using Masson’s trichrome staining, we assessed collagen degradation in periodontal tissues. Similar to results from HE staining, our findings reveal that compared to the control group, periodontitis mice exhibited decreased and sparsely arranged collagen fibers. Furthermore, *C3ar1*^*−/−*^ periodontitis mice showed reduced collagen fiber degradation compared to *C3ar1*^*+/+*^ and *C3ar1*^*+/−*^ genotype periodontitis mice (Fig. 3c, d). These findings suggest that C3a may facilitate dysbiosis-induced hard and soft tissue destruction during periodontitis.

### C3aR deficiency alleviated the expression of proinflammatory cytokines in mice with ligature-induced periodontitis

**Fig. 4:**
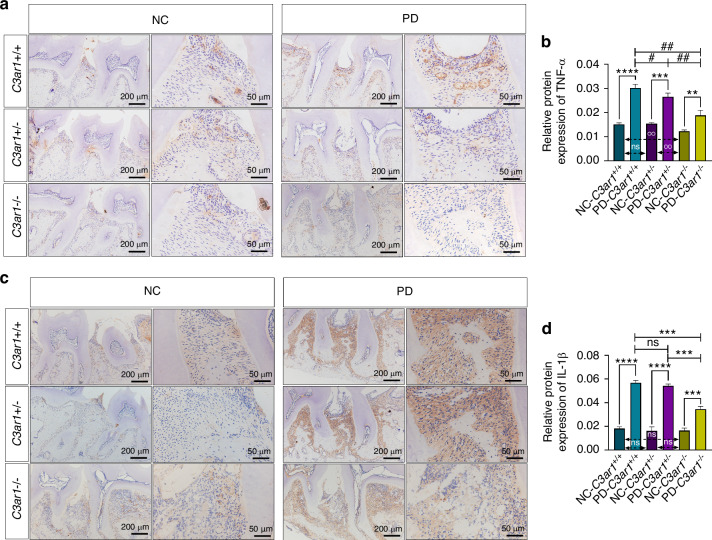
C3a increases the levels of IL-1β and TNF-α in the periodontal tissues of periodontitis mice. **a** TNF-α immunostaining of periodontal tissue between the first and second upper molars. **b** Semiquantitative evaluation and comparison of TNF-α. **c** IL-1β immunostaining of periodontal tissue between the first and second upper molars. **d** Semiquantitative evaluation and comparison of IL-1β. Scale bar, 200 μm and 50 μm. Statistics are shown in mean ± SD (**b**, **d**) accessed by the unpaired *t* test. **P < 0.01; ***P < 0.001; ****P < 0.000 1; ^#^*P* < 0.05; ^##^*P* < 0.01; ns, nonsignificant, respectively

The destruction of periodontal soft and hard tissues is positively correlated with the secretion of inflammatory cytokines.^31^ To investigate the effect of C3aR receptor knockout on the secretion of inflammatory cytokines in periodontal tissues with periodontitis, we performed immunohistochemical staining. The results showed that, compared to the non-ligated side, the protein expression levels of TNF-α (Fig. 4a, b) and IL-1β (Fig. 4c, d) were significantly increased in the ligated side of periodontal tissues across all three genotypes, indicating that ligation enhances the expression levels of inflammatory cytokines in periodontal tissues. However, there were no significant differences in the protein expression levels of TNF-α and IL-1β in the non-ligated side across the three genotypes, suggesting that the C3aR receptor deficiency does not affect the levels of inflammatory cytokines in normal periodontal tissues. Furthermore, the protein expression levels of TNF-α and IL-1β were significantly reduced in the ligated side of *C3ar*^*−/−*^ mice compared to *C3ar*^*+/+*^ and *C3ar*^*+/−*^ mice. Interestingly, *C3ar*^*+/−*^ mice showed decreased levels of the inflammatory cytokine TNF-α in the ligated side compared to *C3ar*^*+/+*^ mice, but there was no significant difference in IL-1β expression. These results indicate that C3aR receptor deficiency can reduce the expression levels of inflammatory cytokines in periodontal tissues with periodontitis, demonstrating that C3a promotes the expression levels of inflammatory cytokines in periodontitis.

### C3a promoted the polarization of macrophages towards the M1 phenotype

**Fig. 5:**
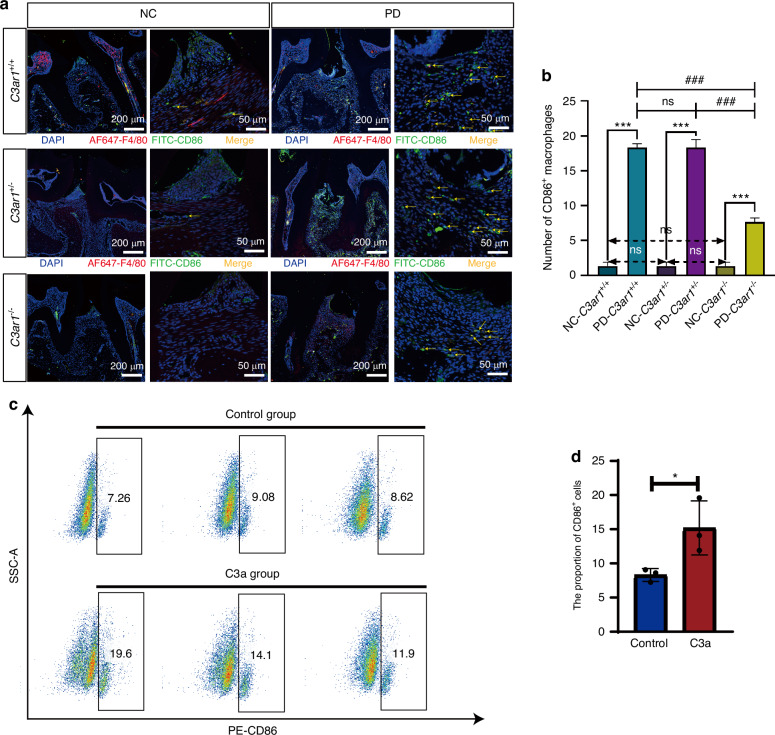
C3a promotes macrophage polarization toward the M1 phenotype. **a** Immunofluorescence staining images of CD86^+^ cells in normal periodontal tissues and periodontitis-affected periodontal tissues from three genotypes of mice. **b** Quantitative analysis of immunofluorescence staining results of periodontal tissues. **c** Flow cytometry results of CD86 expression in RAW274.7 cells treated with and without C3a in vitro. **d** Quantitative analysis of flow cytometry staining results. Scale bar, 200 μm and 50 μm. Statistics are shown in mean ± SD (**b**, **d**) accessed by the unpaired *t* test. *P < 0.05; ***P < 0.001; ^###^*P* < 0.001; ns, nonsignificant, respectively

After identifying the primary cellular sources of C3 and establishing that C3a promotes periodontal tissue destruction, we sought to elucidate the cellular mechanisms by which C3a contributes to this process. We first analyzed single-cell sequencing data from mouse periodontal tissues to determine the major cell types expressing C3aR. Our results revealed that C3aR is predominantly expressed by macrophages (Supplementary Fig. S4a, b). Therefore, we hypothesize that C3a enhances periodontal tissue inflammation by promoting M1 polarization of macrophages. To validate this hypothesis, we conducted immunofluorescence staining experiments to detect the M1 macrophage marker CD86 in the subepithelial fibrous connective tissue of the first and second molars between the ligated and non-ligated sides. The results revealed a significant increase in CD86^+^ cell numbers in the ligated side of periodontal tissues compared to the non-ligated side across all genotypes. Interestingly, periodontitis mice with *C3ar*^*-/-*^ genotype exhibited significantly reduced CD86^+^ cell numbers in periodontal tissues compared to those with *C3ar*^*+/+*^ and *C3ar*^*+/−*^ genotypes. Moreover, there was no significant difference in CD86^+^ cell numbers between *C3ar*^*+/+*^ and *C3ar*^*+/−*^ periodontitis mice. These findings indicate that C3aR deficiency reduces macrophage polarization towards the M1 phenotype (Fig. 5a, b). To further demonstrate the role of C3a in promoting M1 polarization of macrophages, we treated RAW264.7 cell line with recombinant C3a for 12 h in vitro. The results showed a significant increase in the proportion of CD86^+^ cells in the C3a-treated group compared to the control group (Fig. 5c, d). These results collectively demonstrate that the C3a promotes M1 polarization of macrophages.

### C3a promoted osteoclast differentiation

**Fig. 6:**
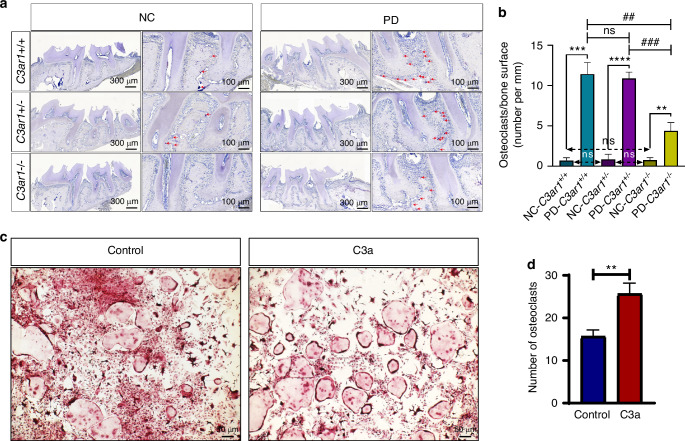
C3a promotes osteoclast differentiation. **a** TRAP staining results of osteoclasts in normal and periodontitis-affected periodontal tissues from three genotypes of mice. **b** Quantitative analysis of TRAP staining results in mouse periodontal tissues. **c** TRAP staining results of osteoclasts induced in vitro, with and without C3a treatment. **d** Quantitative analysis of TRAP staining results of osteoclasts induced in vitro. Scale bar, (TRAP for tissue) 300 μm and 100 μm, (TRAP for cell) 50 μm. Statistics are shown in mean ± SD (**b**, **d**) accessed by the unpaired *t* test. **P < 0.01; ***P < 0.001; ****P < 0.000 1; ^##^*P* < 0.01; ^###^*P* < 0.001; ns, nonsignificant, respectively

Since C3aR is predominantly expressed on the surface of monocyte-macrophages, which can differentiate into osteoclasts, the primary cells mediating alveolar bone loss in periodontitis, we hypothesize that the C3a promotes osteoclast differentiation. To test this hypothesis, we performed TRAP staining on periodontal tissue sections from healthy and periodontitis mice of three different genotypes. The results showed a significant increase in the number of osteoclasts in the ligated side of periodontal tissues in all genotypes compared to the non-ligated side, indicating that periodontal ligation increases the number of osteoclasts in periodontal tissues. There was no significant difference in the number of TRAP^+^ osteoclasts in the non-ligated side across all genotypes, suggesting that C3aR deficiency does not affect the number of osteoclasts in healthy periodontal tissues. Consistent with our hypothesis, the number of osteoclasts in the ligated side of periodontal tissues was significantly reduced in *C3ar*^*-/-*^ mice compared to heterozygous and wild-type periodontitis mice. There was no significant difference in the number of osteoclasts between heterozygous and wild-type mice in the ligated side of periodontal tissues (Fig. 6a, b). These findings indicate that C3aR deficiency reduces osteoclast differentiation. To further demonstrate that C3a promotes osteoclast differentiation in vitro, we treated mouse bone marrow mononuclear cells with C3a in the presence of M-CSF and RANKL. The results showed an increase in the number of osteoclasts in the C3a-treated group compared to the control group (Fig. 6c, d). These findings collectively demonstrate that the C3a promotes osteoclast differentiation.

### C3a promotes M1 macrophage polarization and osteoclast differentiation through the NF-κB pathway

**Fig. 7:**
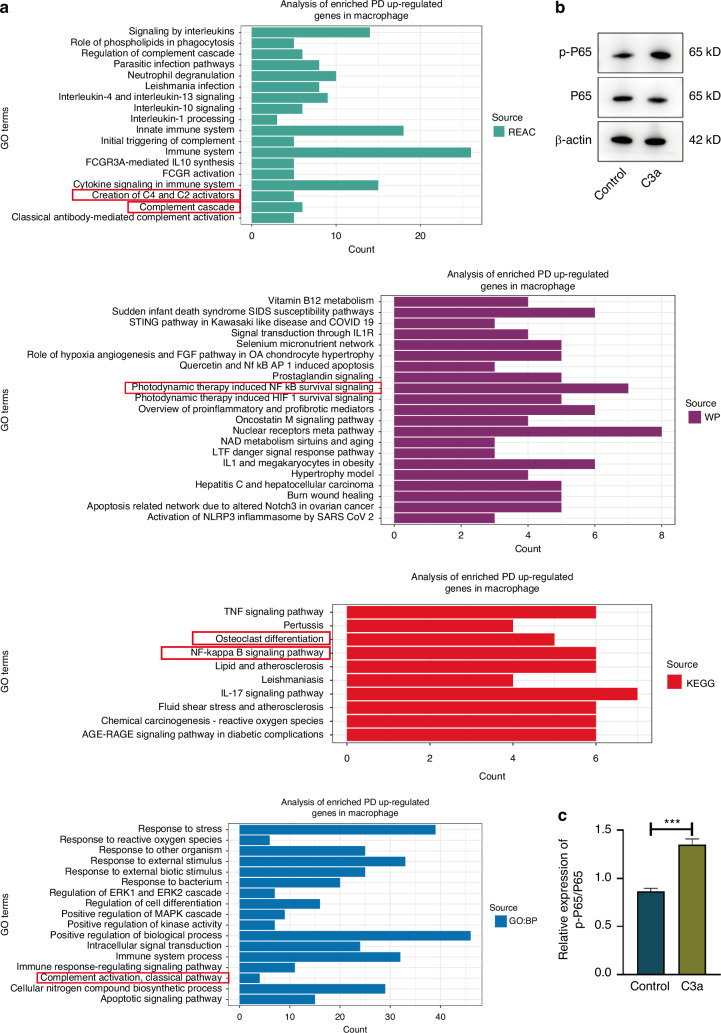
C3a promotes M1 macrophage polarization and osteoclast differentiation through the NF-κB pathway**. a** Enrichment analysis of differentially expressed genes highly expressed in macrophages from the periodontitis group in the REAC database, WP database, KEGG database and GO database. **b** Western blot results of the NF-κB pathway. **c** Quantitative analysis of the Western blot results of the NF-κB pathway. Statistic are shown in mean ± SD (**c**) accessed by the unpaired *t* test. ***P < 0.001

To investigate the signaling pathways through which C3a promotes macrophage polarization and osteoclast differentiation, we performed pathway and functional enrichment analysis using GO, KEGG, WP and REAC databases on the differentially expressed genes in macrophages from periodontal tissue of the periodontitis group compared to those from the control group, based on single-cell sequencing data. The results revealed that the genes highly expressed in macrophages from the periodontitis group were predominantly enriched in functions related to the complement system and the NF-κB pathway (Fig. 7a). To validate these enrichment results, we treated Raw264.7 cells with C3a and assessed the activation of the NF-κB pathway using Western blotting. The results confirmed that C3a indeed activates the NF-κB pathway (Fig. 7b, c). These findings suggest that C3a promotes macrophage polarization and osteoclast differentiation through the NF-κB pathway.

### The findings from mouse studies were also applicable to humans

**Fig. 8:**
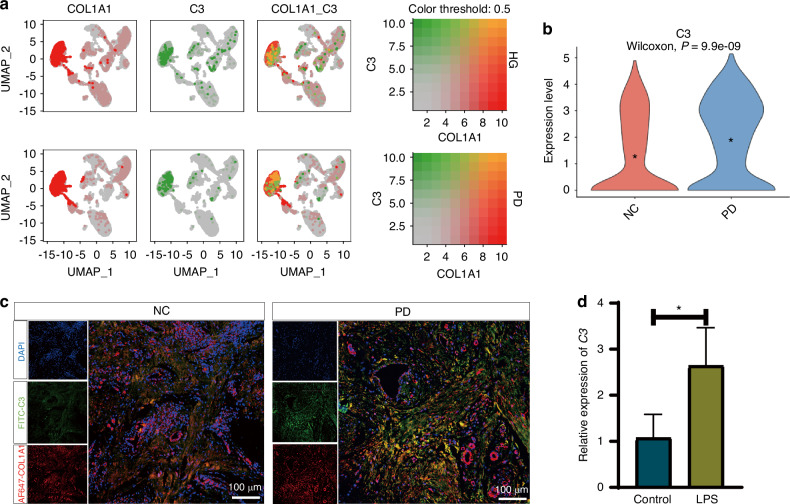
Periodontal fibroblasts are the main source of complement C3 in human. **a** Analysis results of the co-expression of fibroblast marker *COL1A1* and complement *C3*. **b** Violin plot of the C3 gene between the NC and PD groups. **c** Immunofluorescence co-staining results of fibroblast marker COL1A1 and complement C3 in the healthy human and periodontal tissue. Scale bar 100 μm. **d** PCR results of *C3* gene expression in human gingival fibroblasts with or without LPS stimulation. Scale bar, 100 μm. Statistics are shown in mean ± SD (**d**) accessed by the unpaired *t* test. *P < 0.05

Having established that the C3a promotes soft and hard tissue destruction in periodontitis mice through macrophage M1 polarization and osteoclast differentiation, we sought to investigate whether the expression patterns of C3 and C3ar in periodontal tissues of healthy humans and patients with periodontitis, as well as changes in the proportions of various cell types, are similar to those observed in mice. This exploration aims to better apply our research findings to clinical settings. To address this question, we downloaded single-cell transcriptomic sequencing data of periodontal tissues from healthy individuals and periodontitis patients from the GEO database. Through data processing steps including quality control and dimensionality reduction, the entire cell population was classified into 8 cell clusters based on established classical cell markers (Supplementary Fig. S6d): fibroblasts, endothelial cells, T cells, plasma cells, epithelial cells, macrophages, B cells and mast cells (Supplementary Fig. S6a, 6b). Analysis of the proportions revealed that compared to the normal group, the periodontitis group showed increased proportions of plasma cells, macrophages and T cells, and decreased proportions of fibroblasts, endothelial cells and epithelial cells, with minimal changes in B cells and mast cells proportions (Supplementary Fig. S6c). Gene expression analysis of C3 and C3aR revealed that fibroblasts were the primary cell type expressing C3 and macrophages were the primary cell type expressing C3aR in both normal and periodontitis groups. Interestingly, compared to the normal group, periodontitis group exhibited significantly increased expression levels of C3 in fibroblasts and C3aR in macrophages within the periodontal tissues (Fig. 8a, b and Supplementary Fig. S4c, d). At the same time, we validated in vivo, using immunofluorescence co-staining, that the fibroblast-derived C3 levels were higher in the periodontitis group compared to the normal group (Fig. 8c). Additionally, we verified in vitro through RT-qPCR that LPS stimulation increased the C3 gene expression in human gingival fibroblasts (Fig. 8d). These results suggest that our findings derived from periodontitis mice are similarly applicable to humans, highlighting the relevance of our research in clinical contexts.

## Discussion

Given that the soft tissue damage and alveolar bone loss caused by periodontitis are primarily due to an excessive immune-inflammatory response triggered by microorganisms, exploring the pathogenic mechanisms of periodontitis from an immunological perspective, in addition to controlling plaque, may offer promising avenues for effective treatment.^32^ The complement system, as a critical component of the immune system, serves as the first line of defense against pathogenic microorganisms and bridges innate and adaptive immunity.^33^ Therefore, it is likely to play a significant role in the onset and progression of periodontitis induced by periodontal pathogens. In this study, we employ single-cell transcriptome analysis complemented by rigorous experimental validation to elucidate that the hydrolysis product of the central component of the complement system, C3a, participates in the onset and progression of periodontitis by promoting macrophage M1 polarization and osteoclast differentiation. It concludes by suggesting the potential viability of treating periodontitis by inhibiting the function of C3a or reducing local C3a levels in periodontal tissues.

The complement system, an integral part of the immune system, has been implicated in the pathogenesis and progression of periodontitis. Early clinical studies have demonstrated significantly higher levels of complement activation products in the gingival tissue and gingival crevicular fluid of patients with periodontitis compared to healthy individuals.^34^ The induction of experimental gingivitis in human volunteers resulted in progressive complement activation, correlating with increased clinical inflammation.^35^ Conversely, successful periodontal treatment that resolved clinical inflammation inhibited complement activation in the gingival crevicular fluid of treated patients.^36^ Specifically, interception of the complement cascade at its central component, C3, using a locally administered small peptidic compound (Cp40/AMY-101) protected non-human primates from induced or naturally occurring periodontitis.^37^ Consistent with these findings, our study also found that the protein level of complement C3a in the serum of periodontitis mice was significantly higher than that in non-periodontitis mice. This suggests that complement C3a plays a crucial role in the development and progression of periodontitis.

Complement has been considered mainly existing in the systemic compartment, with serum levels of most components of the complement system, including C3, C4, and MBL, being produced by hepatocytes.^38^ Other tissues also contain cells capable of complement production; for example, endothelial and epithelial cells can secrete various complement components, thereby contributing to local processes of diseases.^39^ Additionally, there is a growing body of evidence that local secretion of complement proteins plays an important role in regulating physiological processes even in the absence of further complement activation.^40^ Our findings also support this view. Through single-cell sequencing data analysis of healthy and periodontitis tissues in humans and mice, we discovered that periodontal fibroblasts are the primary source of local C3 in periodontitis. Upon activation of the complement system, C3 is hydrolyzed into C3a and C3b. C3b promotes phagocytosis of bacteria by binding to corresponding receptors on phagocytic cells, or forms C5 convertase complexes with other complement components to participate in the complement cascade reaction. C3a is released and binds to its specific receptor, C3aR, on cell surfaces to exert its effects. Research indicates that numerous cell types express C3a and its receptor C3aR, including various immune cells (such as monocytes, neutrophils, macrophages, T cells, B cells, and mast cells), osteoblasts, osteoclasts, and their precursor cells.^41^ Among these, macrophages are the primary cell type expressing C3a and C3aR within immune cells.^42^ Indeed, in our study, we found that macrophages are the main cell type expressing C3aR. Notably, after the onset of periodontitis, the expression of the C3 gene in periodontal fibroblasts and the C3ar1 gene in macrophages both significantly increased. Furthermore, the complement system’s hydrolytic pathways are enhanced, as evidenced by our single-cell data cell ratio analysis revealing a significant increase in plasma cell secretion of antibodies in the periodontitis group. This augmentation potentially strengthens the pathway involving complement activation by antigen-antibody complexes, thereby leading to increased hydrolysis of C3 into C3a. This is further underscoring the importance of C3a in the pathogenesis and progression of periodontitis.

The interaction between C3a and its receptor C3aR mediates a series of biological effects. For instance, this interaction regulates ATP efflux and promotes the activation of the NOD-like receptor protein 3 (NLRP3) inflammasome by increasing the phosphorylation of extracellular signal-regulated protein kinases 1/2 (ERK1/2), leading to the secretion of the inflammatory cytokine IL-1β by human monocytes, macrophages and dendritic cells.^43^ Additionally, C3a promotes mast cell degranulation and chemokine secretion through the regulation of Ca2^+^ release, playing a role in hypersensitivity reactions.^44^ This axis also facilitates skeletal muscle repair after injury by chemotactically recruiting macrophages to the site of muscle damage.^45^ Furthermore, the C3a promotes M1 macrophage polarization through signaling pathways involving ERK1/2, nuclear factor-κB (NF-κB), and signal transducer and activator of transcription 1 (STAT1), accelerating renal fibrosis in a mouse model of unilateral ureteral obstruction.^19^ In line with these findings, our study demonstrates that the C3a increases the number of M1 macrophages and the expression of M1 macrophage markers in periodontal inflammatory tissues. Furthermore, it was confirmed that C3a effectively activates the NF-κB pathway in macrophages.This further substantiates the role of the C3a in regulating macrophage polarization and suggests that it may mediate periodontal inflammation and soft tissue destruction by modulating macrophage polarization. These findings suggest that the heightened C3a level in periodontitis patients is more than a biomarker of disease severity, but rather reflects a critical destructive factor.

Given that a primary characteristic of periodontitis is alveolar bone loss, and osteoclasts are the only cells in periodontal tissues capable of causing this bone destruction, investigating the reasons behind the increased presence of osteoclasts in periodontal tissues is crucial. Research indicates that M1 macrophages secrete various inflammatory cytokines, such as IL-6, IL-1, TNF-α, and prostaglandin E2 (PGE2), which stimulate osteoclast differentiation and activation, thereby contributing to bone resorption.^46^ Additionally, M1 macrophages can induce the differentiation of Th17 cells, which express high levels of RANKL, further promoting osteoclastogenesis and alveolar bone destruction.^47^ Our findings show that knockout of C3aR decreases the number of M1 macrophages and mitigates alveolar bone loss in mice with periodontitis. This supports the hypothesis that the C3a may regulate osteoclast activity and subsequent alveolar bone destruction by modulating macrophage polarization. Furthermore, our research provides insight into another mechanism where the C3a directly promotes monocyte differentiation into osteoclasts. Recent studies in bone immunology have corroborated our findings. Anita Ignatius et al.‘s research found that C3a and C5a alone were insufficient to induce the release of inflammatory cytokines IL-6 and IL-8 from osteoblasts. However, co-stimulation with the pro-inflammatory cytokine IL-1β significantly enhanced IL-6 and IL-8 expression, along with RANKL and osteoprotegerin (OPG) expression, suggesting that complement may modulate the inflammatory response of osteoblasts in a pro-inflammatory environment.^48^ This interaction may influence osteoblast-osteoclast interactions and osteoclastogenesis, even in the absence of RANKL and M-CSF, thus indicating a direct regulatory role for complement in osteoclast formation.

Our study partially elucidates that the C3a exacerbates periodontitis by promoting macrophage polarization and osteoclast differentiation. However, there are certain limitations to our research. Firstly, since our transgenic mice are global C3aR knockouts and we did not utilize myeloid cell-specific C3aR knockout mice, it is not entirely clear whether the amelioration of periodontitis phenotypes observed in C3aR knockout mice can be solely attributed to macrophage polarization and osteoclast differentiation. Secondly, inflammatory cytokines produced by M1-polarized macrophages can also induce osteoclast differentiation. Therefore, it remains uncertain whether the reduced alveolar bone destruction observed in C3aR knockout mice is primarily due to the decreased number of osteoclasts directly caused by the absence of C3a or the reduced inflammatory cytokine production by macrophages. Thirdly, we did not further elucidate the detailed mechanisms by which the C3a promotes M1 polarization and osteoclast differentiation.

## Conclusion

In conclusion, this study demonstrates through imaging and histological analysis of C3ar knockout mice with periodontitis that fibroblasts derived C3 may mediate periodontal tissue inflammation and alveolar bone loss by promoting macrophage polarization toward the M1 phenotype and osteoclast differentiation. This confirms that, as a frontline defense against periodontal pathogens, the complement system and the excessive immune-inflammatory response mediated by M1 macrophages and bone immune homeostasis accelerate the progression of periodontitis. These findings suggest the potential of targeting complement C3a for periodontitis treatment. However, in the periodontal microenvironment, the activation and function of the complement system, macrophage polarization and osteoclast differentiation are regulated by multiple factors. Further research is needed to better understand how to modulate their immune responses to effectively enhance their protective roles.

## Materials and methods

### Animals

Heterozygous C3ar1 gene knockout mice (background: C57BL/6N) were purchased from Jiangsu Jicui Yaokang Biotechnology Co., Ltd. All animal work was conducted in accordance with the guidelines of the Laboratory Animal Ethics Committee, School of Basic Medical Sciences, Jilin University, China, and adhered to ethical standards as per license number 2024317. The mice were provided with unrestricted access to food and water and housed in a specific pathogen-free (SPF) environment, maintained at a stable temperature of 22 °C and 55% ± 10% humidity, under a consistent 12-h light-dark cycle. To confirm the C3aR deficiency at the genetic level, genotyping PCR was performed using a common forward primer in combination with either a WT-specific reverse primer or a KO-specific primer, following the manufacturer’s instructions for the Red Taq PCR Ready Mix (Sigma-Aldrich).

### Establishment of periodontitis model

Eighteen mice, aged 8-10 weeks, were utilized for ligation experiments, including C3ar1 heterozygous mice (*n* = 6), C3ar1 homozygous mice (*n* = 6), and wild-type (WT) mice (*n* = 6). All mice were first anesthetized by intraperitoneal injection of a mixture containing 66.7 mg/kg ketamine and 6.7 mg/kg xylazine. Subsequently, the maxillary second molar on the left side was ligated with 4-0 polyamide silk, with knots placed on the buccal side between the first and second molars. The contralateral side was left without a ligature to serve as a control. The ligature was maintained for 14 days.

### Micro-CT

After 8 days ligation, all mice were sacrificed by cervical dislocation. The maxillae were harvested and fixed in 4% paraformaldehyde solution for 24 h. The maxillae were then collected and scanned using a micro-CT system (µCT50, Scanco Medical AG, Bassersdorf, Switzerland). The scanning parameters were as follows: 70 kVp, 200 µA, voxel size 34.4 µm, and exposure time 270 ms. Three-dimensional images were reconstructed and analyzed using the manufacturer’s software (SkyScan). The distance between the cementoenamel junction (CEJ) and the apex of the alveolar bone crest (ABC) was measured at six anatomical sites of the maxillary molars, as indicated by the red line segment in Fig. 1a. Additionally, bone-related parameters, including bone volume fraction (bone volume/tissue volume, BV/TV, %), were measured to investigate the variations in bone mass and mineralization between the maxillary first and second molars using the software (SkyScan).

### Hematoxylin-Eosin (HE) staining

After being scanned by micro-CT, the samples were decalcified in 10% EDTA for one month, with the EDTA solution refreshed weekly. The samples were then dehydrated through a graded series of ethanol, embedded in paraffin, and sectioned at a thickness of 4 µm. Subsequently, the sections were routinely dewaxed and rehydrated. Hematoxylin staining solution was applied for 5 min, followed by rinsing with distilled water to remove excess stain. Differentiation was carried out using a differentiation solution for 60 s, with two rinses in tap water, each lasting 3 min. Eosin staining solution was then applied for 2 min, followed by rinsing with distilled water to remove excess stain. The sections were rapidly dehydrated through a graded series of ethanol, cleared in xylene, and mounted with neutral balsam (Solarbio, G1120). Next, neutrophils were distinguished based on their segmented nuclei and rod-shaped morphology, followed by counting and quantitative analysis.

### Masson’s trichrome staining

The slides were dewaxed and rehydrated. They were then immersed in a mordant staining solution and incubated in a water bath at 60 °C for 1 h, followed by rinsing under running water for 10 min. Celestine Blue solution was applied to the sections and stained for 3 min. The slides were gently washed twice with distilled water, each wash lasting 15 s. Mayer’s hematoxylin staining solution was used for dropwise staining for 2 to 3 min, followed by washing with distilled water twice, each wash lasting 10 to 15 s. The sections were counterstained with acid alcohol differentiation solution, followed by rinsing under running water for 10 min. Ponceau-acid fuchsin solution was applied dropwise for 10 min, followed by two washes with distilled water, each wash lasting 10 to 15 s. The slides were then incubated with phosphoric acid solution for approximately 10 min, followed by the addition of aniline blue staining solution for 2 min. This was followed by a wash with a weak acid solution and an additional 2-min incubation. The slides were subsequently dehydrated with 95% ethanol and absolute ethanol, cleared in xylene, and sealed with resin (Solarbio, G1346).

### Immunohistochemical staining

Paraffin sections were deparaffinized and rehydrated, followed by rinsing under running water and soaking in PBS three times for 3 min each. After removing PBS from the sections, an appropriate amount of immunohistochemical antigen retrieval solution (pepsin method, Maixin, DIG-3009) was applied and incubated at 37 °C for 30 min, followed by soaking in PBS three times for 3 min each. The immunostaining procedure was carried out according to the standard steps of the UltraSensitive™ SP (Rabbit) IHC Kit (Maixin, KIT-9706). Specifically, after removing the PBS solution, endogenous peroxidase blocker was added and incubated at room temperature for 10 min, followed by soaking in PBS three times for 3 min each. After removing PBS, a non-specific staining blocker was added and incubated at room temperature for 10 min. The non-specific staining blocker was then removed, and TNF-α (17590-1-AP, Proteintech, 1:200) and IL-1β (YT2322, Immunoway, 1:200) antibodies were applied, followed by incubation overnight at 4 °C. The next day, the sections were equilibrated at room temperature for 30 min, followed by soaking in PBS three times for 3 min each. After removing PBS, biotin-labeled goat anti-mouse IgG polymer was added and incubated at room temperature for 10 min. The sections were then soaked in PBS three times for 3 min each. Streptavidin-biotin complex-peroxidase was added and incubated at room temperature for 10 min, followed by soaking in PBS three times for 3 min each. After removing PBS, DAB chromogen solution (Maixin, DAB-0031) was added and incubated at room temperature for 5 min, followed by rinsing under running water, counterstaining with hematoxylin, dehydrating through a graded series of ethanol, clearing in xylene, and mounting with neutral balsam. The staining intensity of each section was calculated by measuring the optical density using Image Pro Plus.

### Single cell RNA-seq data analysis

The sequencing data for periodontal tissues from healthy and periodontitis mice were acquired from the GSE228635^49^ and GSE184938 datasets. Similarly, sequencing data for periodontal tissues from healthy individuals and periodontitis patients were sourced from the GSE164241^50^ dataset. Initially, single-cell sequencing data from both healthy and periodontitis tissues were harmonized using the Harmony algorithm. Subsequently, cell clusters were annotated based on the significant differential expression of genes across the clusters, identified using the FindAllMarkers function in the Seurat package.

### Immunofluorescent staining

Paraffin sections were routinely deparaffinized to water, followed by antigen retrieval using a pepsin antigen retrieval solution. Non-specific antigen blocking was performed by incubating the sections with 10% FCR blocker in 5% BSA for 60 min at room temperature. Subsequently, tissue sections were incubated with primary antibodies: anti-mouse F4/80-AF647 (151003, Biolegend, 1:100), CD86 (13395-1-AP, Proteintech, 1:200), COL1A1 (ab316222, Abcam, 1:200) and C3 (66157-1-Ig, Proteintech, 1:200) at 4 °C overnight (approximately 12 h). After washing with PBS to remove excess unbound antibodies, the sections were stained with FITC-labeled goat anti-rabbit and AF647-labeled goat anti-mouse antibodies at room temperature for 2 h. Following antigen labeling, nuclei were stained with DAPI (C0065, Solarbio). Multispectral images of the stained slides were captured using confocal microscopy, providing detailed visualization of the antigens and nuclei.

### Flow cytometry

RAW264.7 cell lines were cultured in vitro. When the cells reached approximately 90% confluence, they were scraped and seeded at a density of 5×10^4^ cells per cm² in a 6-well cell culture plate. Upon reaching 70% confluence, the cells were divided into two groups: a control group and a C3a-treated group. The control group was cultured in normal medium, while the C3a-treated group was cultured in complete medium containing 200 ng/mL of C3a for 24 h. After incubation, the cells were scraped, and residual medium was removed by washing with PBS. Non-specific antigens were blocked by incubating the cells in a 5% BSA solution containing 10% FCR blocker at 4 °C for 10 min. The cells were then incubated with anti-mouse PE-CD86 (105007, Biolegend, 1:100) at 4 °C for 30 min. Unbound antibodies were washed away with flow cytometry buffer. The cells were fixed with 2% paraformaldehyde at room temperature for 10 min, followed by washing with flow cytometry buffer to remove the fixative. The cells were then resuspended in 200 µL of flow cytometry buffer, stained with DAPI, and immediately subjected to flow cytometry analysis. The FACS analysis was performed using a MACSQuant Analyzer 16 flow cytometer (Miltenyi Biotec), and data were analyzed using FlowJo software.

### Histological section TRAP staining

Paraffin sections were deparaffinized to water using standard protocols, followed by fixation with pre-cooled TRAP fixative for 1 min. After rinsing with distilled water, TRAP incubation solution was added and the sections were incubated at 37 °C for 1 h. The sections were then rinsed with distilled water and counterstained with hematoxylin for 2 min, followed by bluing in tap water for 10 min. After a final rinse with distilled water, the sections were mounted using an aqueous mounting medium.

### Osteoclast extraction, culture and TRAP staining

Bone marrow cells were isolated from the tibiae and femurs of 5-week-old mice (C57BL/6N background) and cultured overnight in α-MEM supplemented with 1% penicillin-streptomycin and 10% fetal bovine serum. Bone marrow-derived macrophages (BMMs) were obtained by culturing the non-adherent cells from the bone marrow cell culture with 30 ng/mL M-CSF for 3 days. To obtain pre-osteoclasts (pOCs) and mature osteoclasts, BMMs were further cultured for 4 days in α-MEM complete medium containing 100 ng/mL RANKL and 30 ng/mL M-CSF. In cell treatment experiments, BMMs seeded in 48-well plates were cultured with or without C3a in the presence of RANKL and M-CSF for 3 days. The cultured cells were then fixed with 4% paraformaldehyde (PFA) for 15 min and permeabilized with 0.1% Triton X-100. TRAP staining of the cultured cells was performed using a tartrate-resistant acid phosphatase (Sigma-Aldrich, MO, USA) according to the manufacturer’s instructions. Osteoclast formation was assessed with a light microscope, and TRAP-positive cells containing more than three nuclei were counted as osteoclasts.

### Enzyme-linked immunosorbent assay (ELISA)

Blood samples were collected from both healthy and periodontitis mice. The samples were allowed to clot at room temperature for 20 min and then centrifuged at 2 500 revolutions per minute (rpm) for 20 min to separate the serum. The levels of C3a in these samples were quantitatively determined strictly following the instructions provided with the mouse C3a ELISA kits (ml001969, Enzyme-linked Biotechnology).

Briefly, blank wells, standard wells, and sample wells were set up. Diluted serum samples (1:5) were added to the sample wells (50 µL per well) and incubated at 37 °C for 30 min. Unbound samples were washed away, and then 50 µL of enzyme-labeled reagent was added to each well and incubated at 37 °C for another 30 min. After washing away the unbound enzyme-labeled reagent, 100 µL of chromogenic substrate solution was added to each well and incubated at 37 °C in the dark for 30 min. Subsequently, 50 µL of stop solution was added to each well. The optical density (OD) of each well was measured at 450 nm using a microplate reader within 15 min. A standard curve was plotted from the OD values of the standard wells, and the concentrations of C3a in the sample wells were calculated accordingly.

### RNA extraction and RT-qPCR analysis

Following a 1-day incubation period with or without the P.g LPS, total RNAs of Raw264.7 cells were extracted using the DNAiso Plus reagent (Takara, Chaoyang, Beijing, China). cDNAs were amplified using a reverse transcription kit (YEASEN, Pudong, Shanghai, China). Quantitative reverse transcription PCR (RT-qPCR) was conducted employing Hieff qPCR SYBR Green Master Mix (YEASEN) on a Biorad Real-Time Fluorescent Quantitative PCR Detection System. The expression levels of genes in each sample were normalized to ACTB mRNA as an internal control, and the 2^−ΔΔCt^ method was applied for data analysis. Experiments were repeated at least three times.

### Western blot analysis

Proteins were extracted with PIPA lysis buffer, and lysates were used to run electrophoresis on a 10% Tris-Glycine PAGE Gel before being transferred onto polyvinylidene fluoride (PVDF) membrane. The membrane underwent was block with TBST buffer plus BSA, incubated with primary antibodies at 4 °C overnight. Primary antibodies, rabbit anti-P65 (Cell Signaling Technology), rabbit anti-p-P65 (CST), rabbit anti-P-Stat3 (CST) and mouse anti-Beta Actin (β-actin) (Proteintech) were used in this study. Then, membrane was incubated with horseradish peroxidase-conjugated secondary antibodies, either goat anti-rabbit or goat anti-mouse (Proteintech) for 1 h at room temperature. Detection was achieved using the HPR substrate ECL (Proteintech), and band intensities were quantified with ImageJ software.

### Statistical analysis

Statistical analyses were conducted using GraphPad Prism version 9.0 software, which facilitated the execution of unpaired *t*-tests for comparing differences between two groups. Results were deemed statistically significant if *P* < 0.05, with significance levels denoted as follows: *for *P* < 0.05, ** for *P* < 0.01, and *** for *P* < 0.001.

## Supplementary information

Identification of cell populations in the single-cell sequencing results of periodontitis in human

Identification of cell populations in the single-cell sequencing results of periodontitis in mice

Increased levels of C3a in the serum of mice with periodontitis

Genotype identification results of C3aR-deficient mice

C3aR is primarily expressed by myeloid cells in humans and mice

The expression level of C3 in the periodontitis group epithelial cells was markedly elevated compared to the control group

Supplementary Figures

## Data Availability

Single-cell sequencing data of gingival tissues from healthy individuals and periodontitis patients can be accessed from the GEO database (GSE164241). Single-cell sequencing data of gingival tissues from healthy mice and mice with periodontitis can be accessed from the GEO database (GSE228635 and GSE184938).
