# Author Correction: Fibroblast derived C3 promotes the progression of experimental periodontitis through macrophage M1 polarization and osteoclast differentiation

**DOI:** 10.1038/s41368-025-00383-7

**Published:** 2025-07-02

**Authors:** Feilong Ren, Shize Zheng, Huanyu Luo, Xiaoyi Yu, Xianjing Li, Shaoyi Song, Wenhuan Bu, Hongchen Sun

**Affiliations:** 1 Hospital of Stomatology, Jilin University, Changchun, China; 2 Jilin Provincial Key Laboratory of Tooth Development and Bone Remodeling, School and Hospital of Stomatology, Jilin University, Changchun, China; 3 Jilin Provincial Key Laboratory Oral Biomedical Engineering, Jilin University, Changchun, China

**Keywords:** Mechanisms of disease, Proteolysis

Correction to: *International Journal of Oral Science* 10.1038/s41368-025-00361-z, published online 17 April 2025

Following publication of the original article^1^, the authors regrettably identified the following errors that require correction:

The legend of Fig. 1c should be corrected from:

“**c** Immunofluorescence co-staining results of fibroblast marker COL1A1 and complement C3 in the healthy mouse and periodontal tissue.”

To:

“**c** Immunofluorescence co-staining results of fibroblast marker COL1A1 and complement C3 in healthy mouse periodontal tissue and periodontal tissue from mice with periodontitis.”

The label of Fig. 5b is incorrect, where “Number of CD68^+^ macrophages” should be “Number of CD86^+^ macrophages”. Therefore, Fig. 5 should be corrected from:
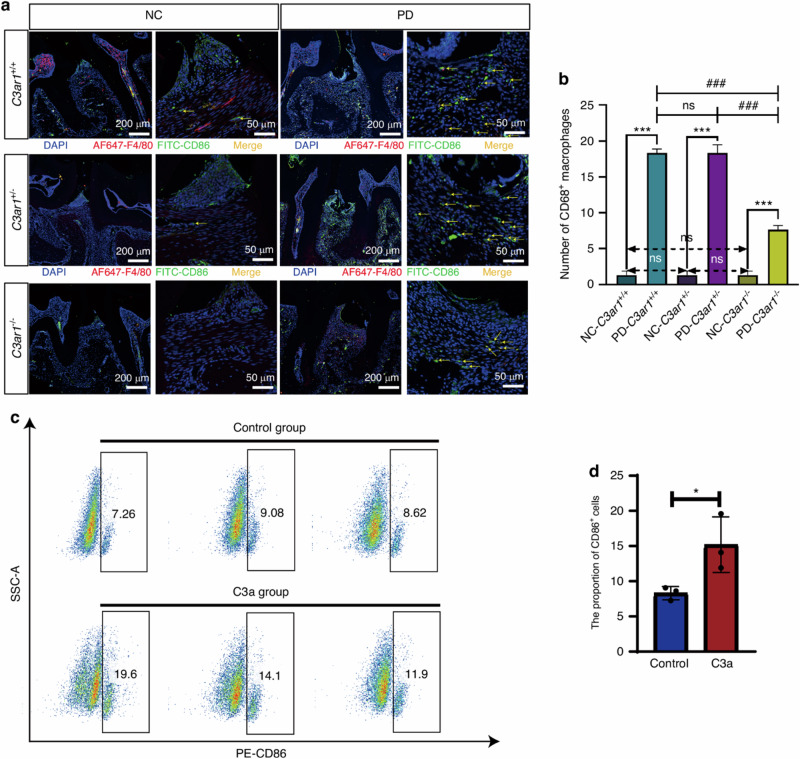


**Fig. 5** C3a promotes macrophage polarization toward the M1 phenotype. **a** Immunofluorescence staining images of CD86^+^ cells in normal periodontal tissues and periodontitis-affected periodontal tissues from three genotypes of mice. **b** Quantitative analysis of immunofluorescence staining results of periodontal tissues. **c** Flow cytometry results of CD86 expression in RAW274.7 cells treated with and without C3a in vitro. d Quantitative analysis of flow cytometry staining results. Scale bar, 200 μm and 50 μm. Statistics are shown in mean ± SD (**b**, **d**) accessed by the unpaired t test. *P < 0.05; ***P < 0.001; ^###^P < 0.001; ns, nonsignificant, respectively

To:
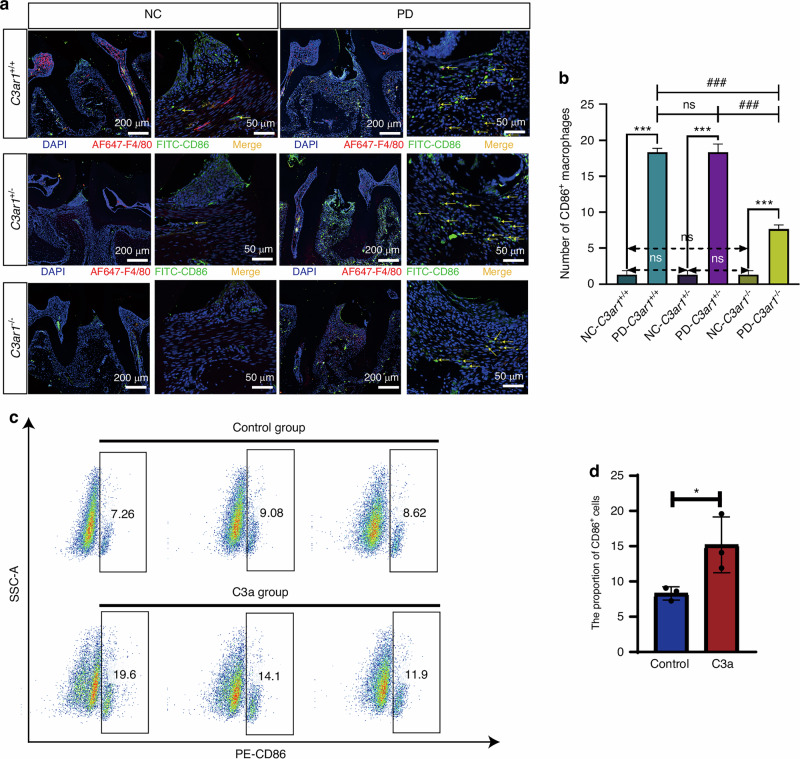


**Fig. 5** C3a promotes macrophage polarization toward the M1 phenotype. **a** Immunofluorescence staining images of CD86^+^ cells in normal periodontal tissues and periodontitis-affected periodontal tissues from three genotypes of mice. **b** Quantitative analysis of immunofluorescence staining results of periodontal tissues. **c** Flow cytometry results of CD86 expression in RAW274.7 cells treated with and without C3a in vitro. d Quantitative analysis of flow cytometry staining results. Scale bar, 200 μm and 50 μm. Statistics are shown in mean ± SD (**b**, **d**) accessed by the unpaired t test. *P < 0.05; ***P < 0.001; ^###^P < 0.001; ns, nonsignificant, respectively

In Materials and methods (Immunofluorescent staining) section, one sentence should be corrected from:

“Subsequently, tissue sections were incubated with primary antibodies: anti-mouse F4/80-AF647 (151003, Biolegend, 1:100), CD86 (13395-1-AP, Proteintech, 1:200), COL1A1 (66288-1-Ig, Proteintech, 1:200) and C3 (66157-1-Ig, Proteintech, 1:200) at 4 °C overnight (approximately 12 h).”

To:

“Subsequently, tissue sections were incubated with primary antibodies: anti-mouse F4/80-AF647 (151003, Biolegend, 1:100), CD86 (13395-1-AP, Proteintech, 1:200), COL1A1 (ab316222, Abcam, 1:200) and C3 (66157-1-Ig, Proteintech, 1:200) at 4 °C overnight (approximately 12 h).”

In Materials and methods (Flow cytometry) section, one sentence should be corrected from:

“The cells were then incubated with anti-mouse PE-CD68 (105007, Biolegend, 1:100) at 4 °C for 30 min.”

To:

“The cells were then incubated with anti-mouse PE-CD86 (105007, Biolegend, 1:100) at 4 °C for 30 min.”

These errors were inadvertent and do not affect the study’s conclusions.

The original article^1^ has been updated.
