# Supplementary figures and images for: Fibroblast derived C3 promotes the progression of experimental periodontitis through macrophage M1 polarization and osteoclast differentiation
Source: Int J Oral Sci. 2025 Apr 17;17:30. doi: 10.1038/s41368-025-00361-z (PMC12003657; doi:10.1038/s41368-025-00361-z)

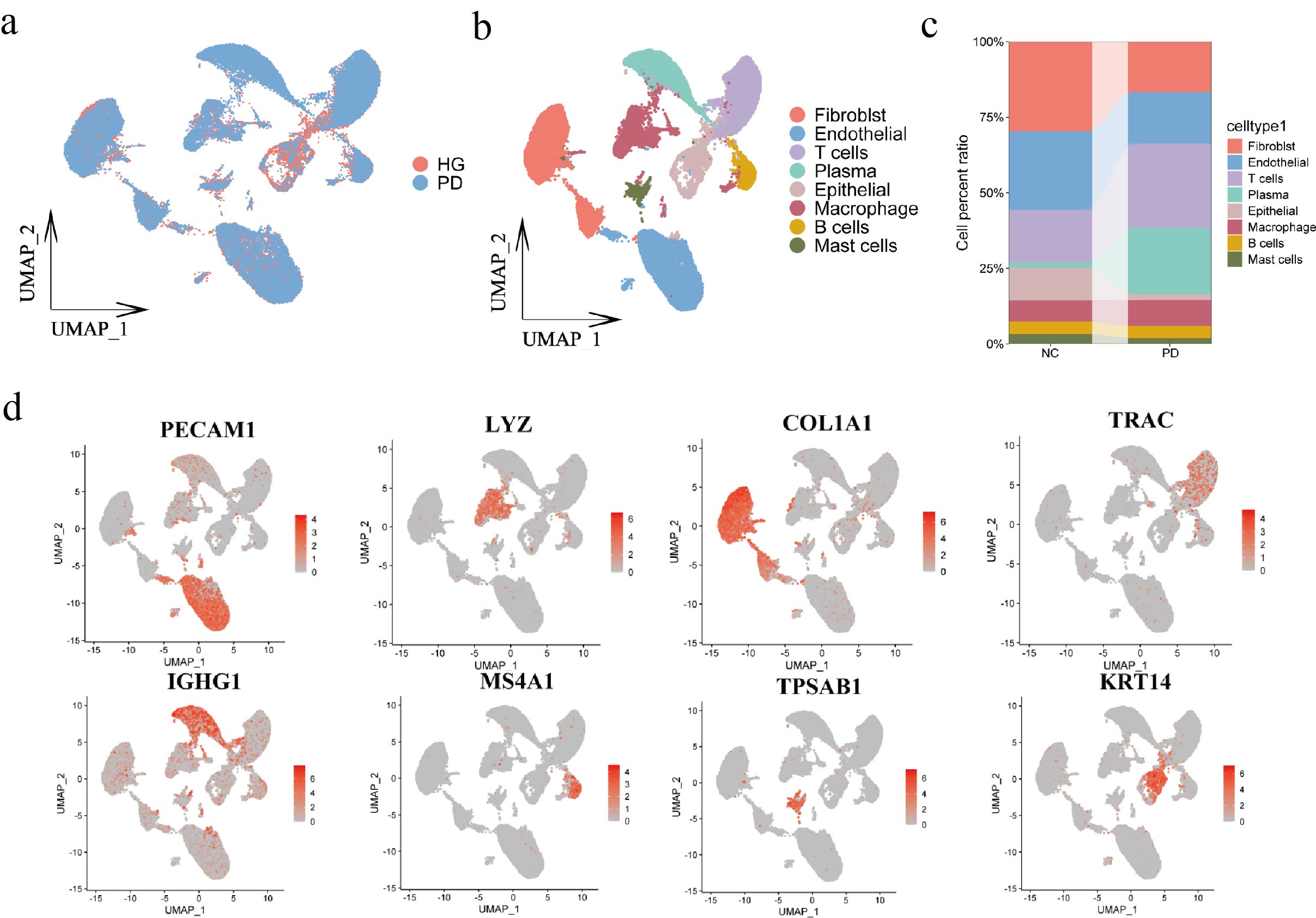

Supplement: Supplementary file 1 — Identification of cell populations in the single-cell sequencing results of periodontitis in human [file 41368_2025_361_MOESM1_ESM.jpg]

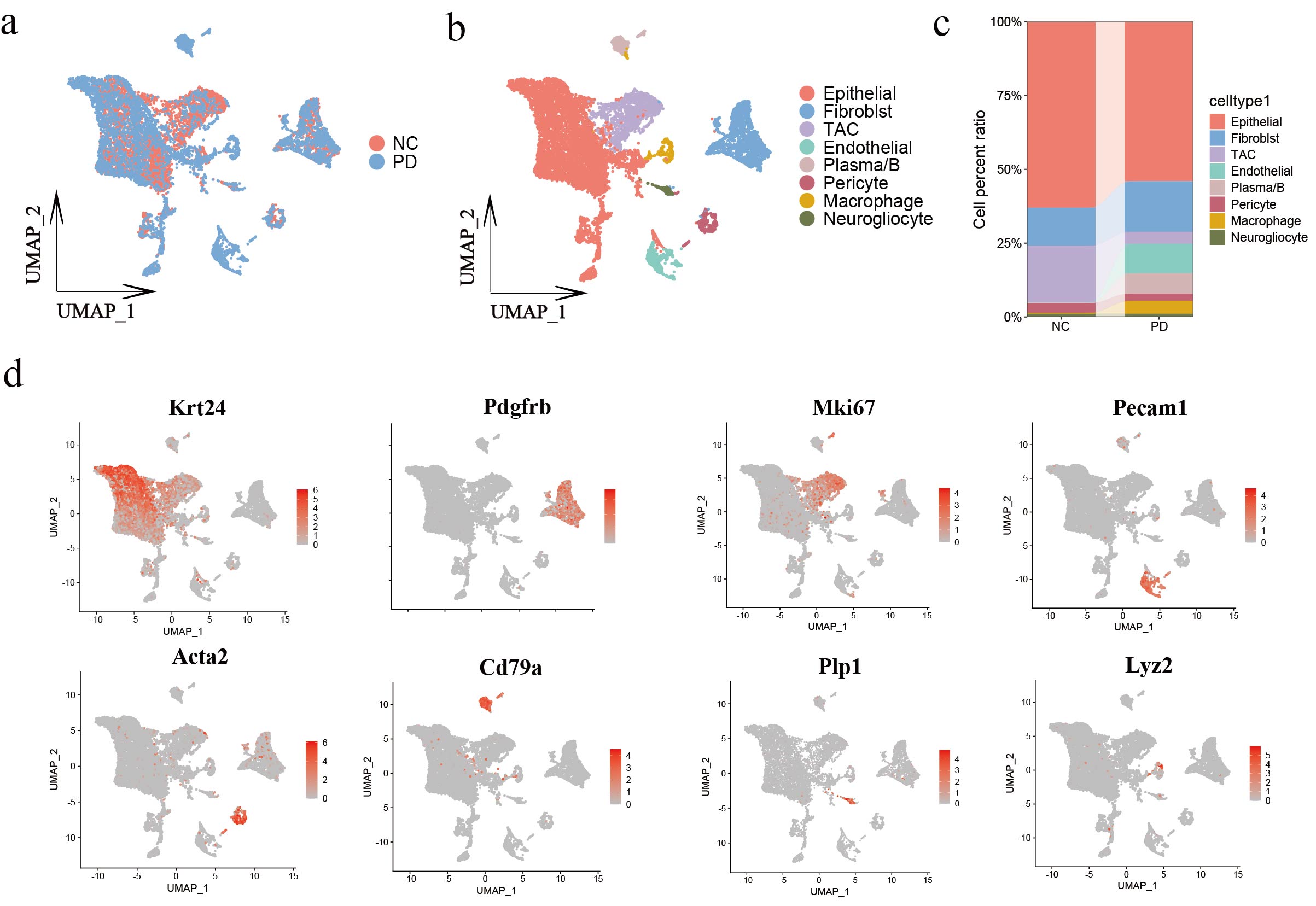

Supplement: Supplementary file 2 — Identification of cell populations in the single-cell sequencing results of periodontitis in mice [file 41368_2025_361_MOESM2_ESM.jpg]

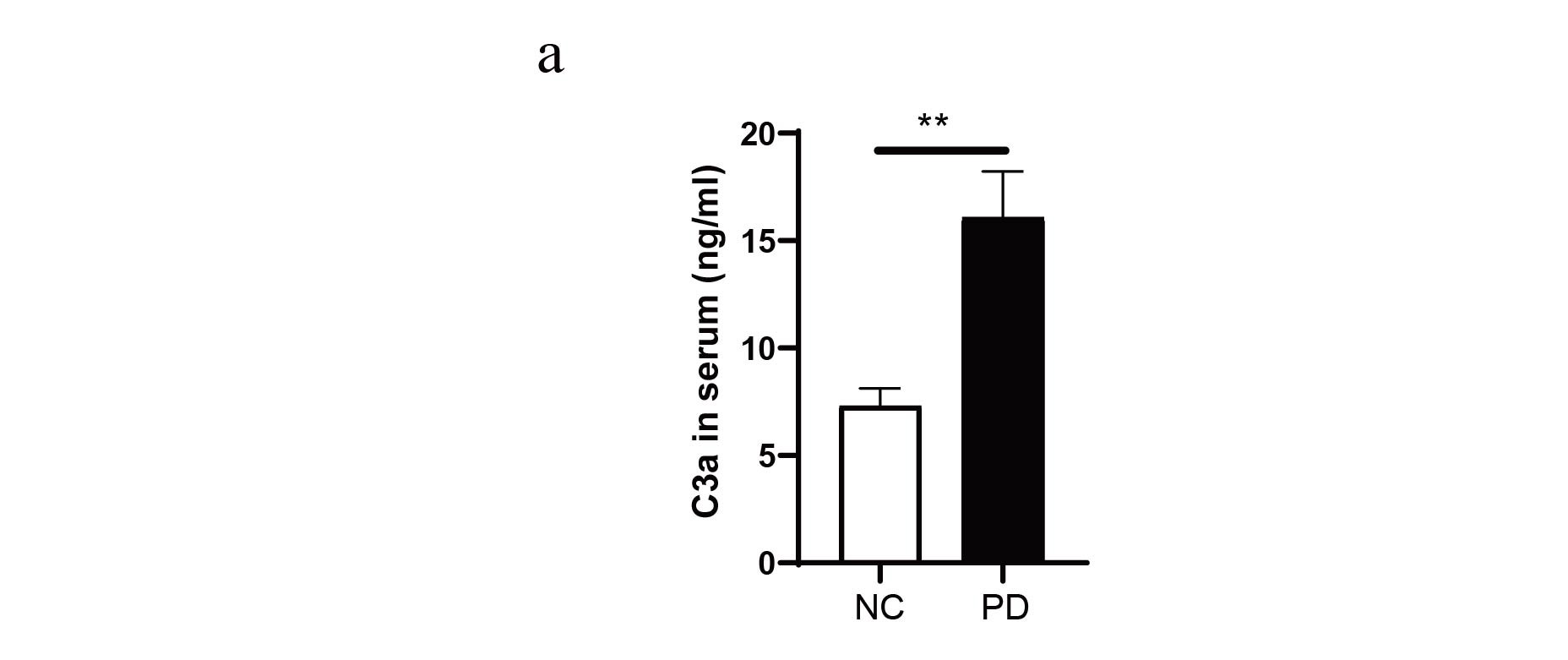

Supplement: Supplementary file 3 — Increased levels of C3a in the serum of mice with periodontitis [file 41368_2025_361_MOESM3_ESM.jpg]

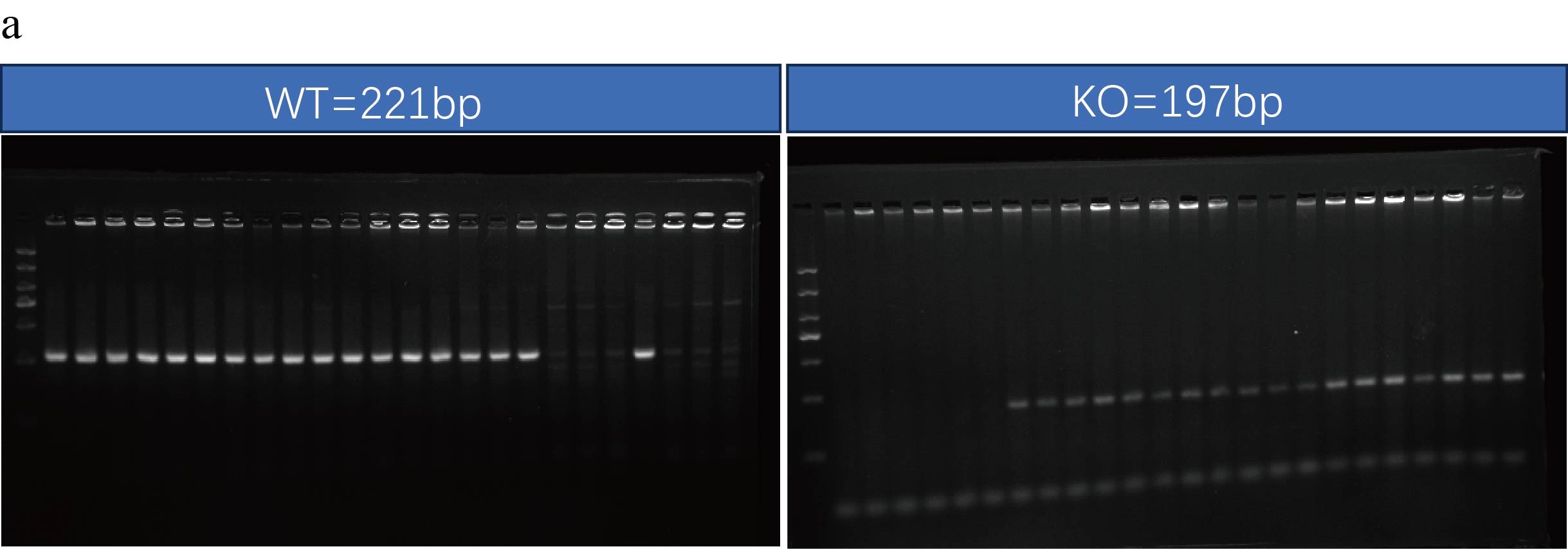

Supplement: Supplementary file 4 — Genotype identification results of C3aR-deficient mice [file 41368_2025_361_MOESM4_ESM.jpg]

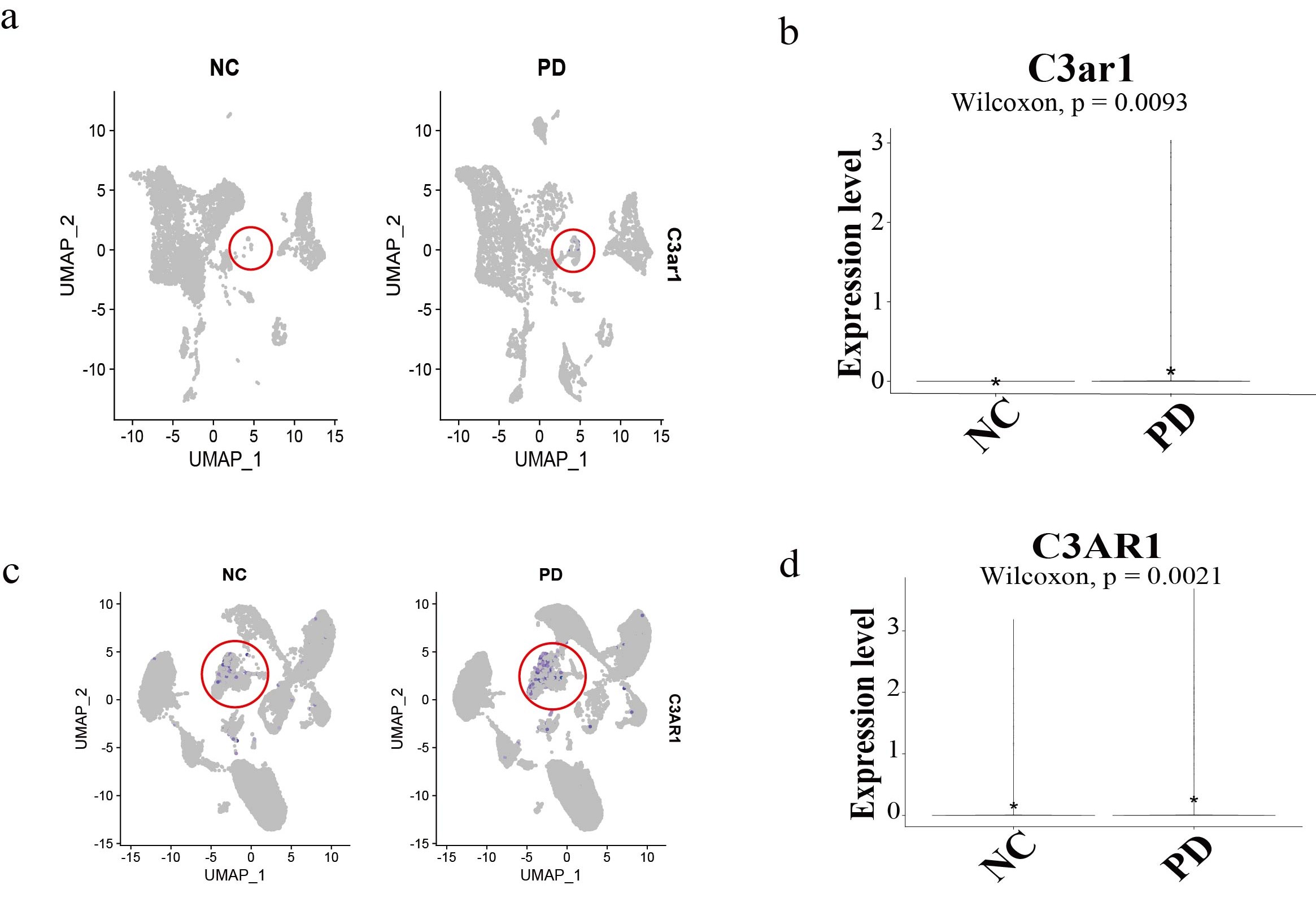

Supplement: Supplementary file 5 — C3aR is primarily expressed by myeloid cells in humans and mice [file 41368_2025_361_MOESM5_ESM.jpg]

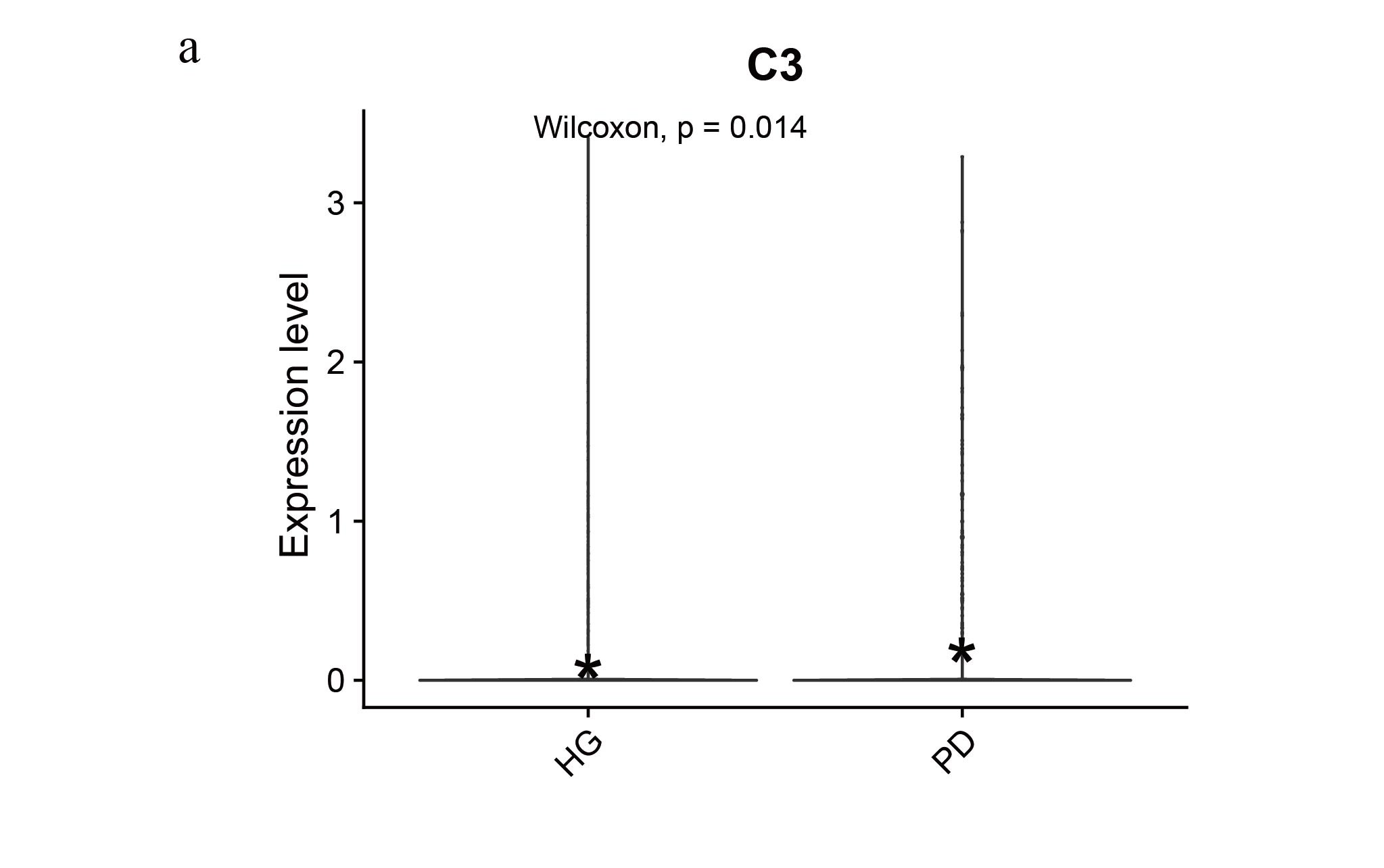

Supplement: Supplementary file 6 — The expression level of C3 in the periodontitis group epithelial cells was markedly elevated compared to the control group [file 41368_2025_361_MOESM6_ESM.jpg]
