# Supplementary material for: Fibroblast derived C3 promotes the progression of experimental periodontitis through macrophage M1 polarization and osteoclast differentiation
Source: Int J Oral Sci. 2025 Apr 17;17:30. doi: 10.1038/s41368-025-00361-z (PMC12003657; doi:10.1038/s41368-025-00361-z)
Supplement: Supplementary file 7 — Supplementary Figures [file 41368_2025_361_MOESM7_ESM.docx]

**Supplementary Figure 1.** Identification of cell populations in the single-cell sequencing results of periodontitis in mice. **a** UMAP plots of dimensional reduction analysis for all cells in the NC and PD groups. **b** Entire periodontal cell population is segregated into 8 distinct clusters using Uniform Manifold Approximation and Projection (UMAP) visualization. **c** Bar graphs comparing the proportions of each cell type between the NC and PD groups. **d** Eexpression patterns of classic markers for 8 cell populations in the single-cell sequencing results of murine periodontitis.

**Supplementary Figure 2.** Increased levels of C3a in the serum of mice with periodontitis. **a** Quantitative analysis of C3a levels in serum from healthy and periodontitis mice using ELISA.

**Supplementary Figure 3.** Genotype identification results of C3aR-deficient mice. **a** Genotype identification gel electrophoresis results for three mouse genotypes.

**Supplementary Figure 4.** C3aR is primarily expressed by myeloid cells in humans and mice. **a** Feature plots showing differential gene expression of *C3ar1* in myeloid cells (as shown in red circle) between normal and periodontitis groups in mouse single-cell data analysis. **b** Violin plots showing differential gene expression of *C3ar1* in myeloid cells between normal and periodontitis groups in mouse single-cell data analysis. **c** Feature plots depicting differential expression of *C3AR1* in myeloid cells (as shown in red circle) between normal and periodontitis groups in human single-cell data analysis. **d** Violin plots depicting differential expression of *C3AR1* in myeloid cells between normal and periodontitis groups in human single-cell data analysis.

**Supplementary Figure 5.** The expression level of *C3* in the periodontitis group epithelial cells was markedly elevated compared to the control group. **a** Represents the average expression level of the *C3* gene in epithelial cells from both groups.

**Supplementary Figure 6.** Identification of cell populations in the single-cell sequencing results of periodontitis in human. **a** UMAP plots of dimensional reduction analysis for all cells in the NC and PD groups. **b** Entire periodontal cell population is segregated into 8 distinct clusters using Uniform Manifold Approximation and Projection (UMAP) visualization. **c** Bar graphs comparing the proportions of each cell type between the NC and PD groups. **d** Expression patterns of classic markers for 8 cell populations in the single-cell sequencing results of human periodontitis.
